# Lacosamide Safety During Pregnancy and Breastfeeding: A Single-Centre Experience and Comprehensive Narrative Review

**DOI:** 10.3390/pharmacy14020058

**Published:** 2026-04-01

**Authors:** Kamila Saramak, Manuela Kaml, Marina Peball, Luisa Delazer, Gerald Walser, Anna Hussl, Iris Unterberger, Alexandra Astner-Rohracher

**Affiliations:** Department of Neurology, Medical University of Innsbruck, 6020 Innsbruck, Austria; kamila.saramak@tirol-kliniken.at (K.S.);

**Keywords:** epilepsy, antiseizure medications, lacosamide, pregnancy, breastfeeding

## Abstract

(1) Background: The management of epilepsy during pregnancy requires balancing effective seizure control against potential teratogenic effects of antiseizure medications (ASMs). Data on the safety of lacosamide (LCM), a third-generation ASM, during pregnancy and breastfeeding are limited. (2) Methods: To evaluate the safety and efficacy of LCM during pregnancy and breastfeeding, we report a single-centre case series and provide a comprehensive narrative review of the literature. (3) Results: In total, 22 cases of maternal exposure to LCM throughout pregnancy (1 monotherapy, 21 polytherapy) were identified, resulting in 21 live births (95.5%). Congenital malformations (atrial septal defect) were observed in one offspring exposed to LCM and levetiracetam (4.8%). Twelve newborns were breastfed (57.1%) without neurodevelopmental delay after twelve months. The literature search identified 16 studies, overall reporting data on 627 pregnancies with LCM (236 monotherapy, 391 polytherapy). Among 632 available pregnancy outcomes (3 twin pregnancies and 1 triplet in the polytherapy group) the proportion of live births was 81.3% (514/632). Major congenital malformations were reported in 2.5% (6/236) with LCM monotherapy and 11.9% (47/396) with polytherapy. (4) Conclusions: According to the literature, no major safety concerns, especially in LCM monotherapy, and no specific malformations associated with LCM exposure were identified. Conclusions are limited by the heterogeneity of studies and the small number of monotherapy-exposed cases. Larger, prospective studies with longer follow-up are required.

## 1. Introduction

Epilepsy affects approximately 0.3–0.8% of pregnant women, making its management during pregnancy and breastfeeding a persistent clinical challenge [1]. Treatment decisions, particularly in refractory cases, require a careful balance between the maternal and foetal risks associated with inadequate seizure control and the potential teratogenic effects of antiseizure medications (ASMs) [2]. In utero exposure to ASMs raises concerns regarding its impacts on foetal growth, major congenital malformations (MCMs), long-term neurocognitive developmental and behavioural outcomes for exposed offspring [3]. Large international pregnancy registries for epilepsy were established more than 20 years ago to assess the risks of MCMs and neurodevelopmental disorders in offspring exposed to ASMs and guide treatment and counselling of women with epilepsy (WWE) during pregnancy and breastfeeding [4]. However, they mainly provide data on “older-generation” ASMs and focus on the impact of monotherapy, whereas data on “newer” ASMs and polytherapy remain scarce.

Current evidence shows that the lowest prevalence of MCMs at one year after birth has been observed in offspring exposed to monotherapy with levetiracetam (LEV) (2.5%), oxcarbazepine (OXC) (2.9%), and lamotrigine (LTG) (3.1%). These rates fall within the range reported for offspring unexposed to ASMs, positioning these drugs among the safer ASM options during pregnancy. In contrast, valproate (VPA) monotherapy is associated with the highest prevalence of MCMs (9.9%) [5] and an up to 5-fold increased risk for neurodevelopmental disorders in the offspring [6,7]. For topiramate (TPM), recent findings also indicate a substantial dose-dependent risk for MCM (6.3%) and an up to 4-fold increased risk for intellectual disability (ID) as well as a 2-fold increased risk for autism spectrum disease (AD) [7,8]. The first-generation sodium channel blocker carbamazepine (CBZ) is similarly linked to an increased prevalence of MCMs (5.5%), with evidence suggesting a dose-dependent increase in MCM [2,5].

Lacosamide (LCM) is a third-generation ASM and acts by enhancing the slow inactivation of voltage-gated sodium channels [9]. Due to its efficacy and favourable tolerability profile, LCM is increasingly prescribed to WWE of childbearing age [10]. However, available data remain insufficient to draw definite conclusions regarding the association between LCM and MCMs, as well as neurodevelopmental outcomes in the offspring [8,11,12]. Given that LCM transfer into breast milk is up to 20%, it is crucial to further investigate the potential short- and long-term adverse effects in breastfed offspring [13,14,15]. Furthermore, especially in women with difficult-to-treat epilepsy, the use of more than one ASM during pregnancy may be necessary to maintain seizure control. The risk of MCM and intrauterine death is overall higher with ASM polytherapy [16], although the choice of ASMs used in combination is the key determinant of teratogenicity [17]. LCM was initially licensed as add-on therapy for focal seizures. Although it is available as monotherapy now, it is still frequently used in complicated cases as part of polytherapy, making its management and counselling throughout pregnancy even more challenging [10].

The aim of this study is to evaluate the safety of LCM use during pregnancy and breastfeeding, and its potential effects on offspring neurodevelopment. In addition, we provide a comprehensive narrative review summarising the current available evidence.

## 2. Materials and Methods

### 2.1. Study Design

This study comprised two components: a single-centre retrospective case series of WWE treated with LCM during pregnancy and breastfeeding, and a comprehensive narrative review of the existing literature.

### 2.2. Retrospective Case Series

#### 2.2.1. Patient Identification

We searched the database of our epilepsy outpatient clinic to identify WWE who were treated with LCM monotherapy or polytherapy throughout pregnancy and/or breastfeeding. Eligible patients were identified through electronic medical records, and all cases meeting these criteria during the study period (2019–2025) were included.

#### 2.2.2. Data Collection

All pregnancies underwent clinical evaluation either in person or by telephone interview at three-month intervals from the beginning of pregnancy until at least twelve months after delivery. During in-person evaluations, serum LCM concentrations were measured. Clinical and obstetric data were retrospectively extracted from medical records and included seizure type and frequency, LCM dosage and plasma concentrations, treatment regimen (monotherapy vs. polytherapy), treatment-related adverse effects, pregnancy course, delivery and breastfeeding, birth outcomes, congenital malformations, and neonatal development according to parental reports. Data on nicotine use, concomitant medication, and folic acid supplementation were also collected.

#### 2.2.3. Statistical Analysis

All statistical analyses were conducted using MATLAB R2022b (The MathWorks, Inc., Natick, MA, USA). Due to the small sample size and binary nature of outcome variables, associations between ASM dosages and adverse pregnancy and neonatal outcomes were assessed using the Mann–Whitney U test. The relationship between breastfeeding and developmental abnormalities was evaluated using Fisher’s exact test. A two-sided *p*-value of ≤0.05 was considered statistically significant for all tests.

#### 2.2.4. Ethical Considerations

Ethics approval was obtained from the local ethics committee (EK 1190/2019).

### 2.3. Comprehensive Narrative Review of the Literature

For this narrative review, supported by a structured literature search, studies reporting exposure to LCM during pregnancy and/or breastfeeding were identified. The search was performed in PubMed, Europe PMC, and the Cochrane Trials database. The following search terms were used: “pregnancy” [MeSH Terms] OR “breastfeeding” [MeSH Terms] OR “teratogenicity” [MeSH Terms], combined with “lacosamide” [Title/Abstract].

Given the limited number of studies on well-characterised pregnancies exposed to LCM—most of which consist of case reports, case series, and observational studies—alongside larger datasets derived from less precisely defined patient populations, the available evidence is heterogenous. Accordingly, a descriptive synthesis of the available data was performed instead of a formal systematic analysis, with careful consideration of the strengths and limitations of individual studies.

Original studies and case reports conducted in humans and published in English that prospectively or retrospectively reported outcomes of LCM used as monotherapy or polytherapy, including foetal loss, major and minor congenital malformations, and child development, were included. Apart from the requirement of peer review, no formal risk-of-bias or methodological quality assessment was performed, consistent with the narrative nature of this review. However, limitations of individual studies are discussed in the Results Section. Reference lists of all eligible articles were manually screened to identify additional potentially relevant studies. No time restrictions were applied, and the final search was conducted on 1 January 2026.

**Figure 1:**
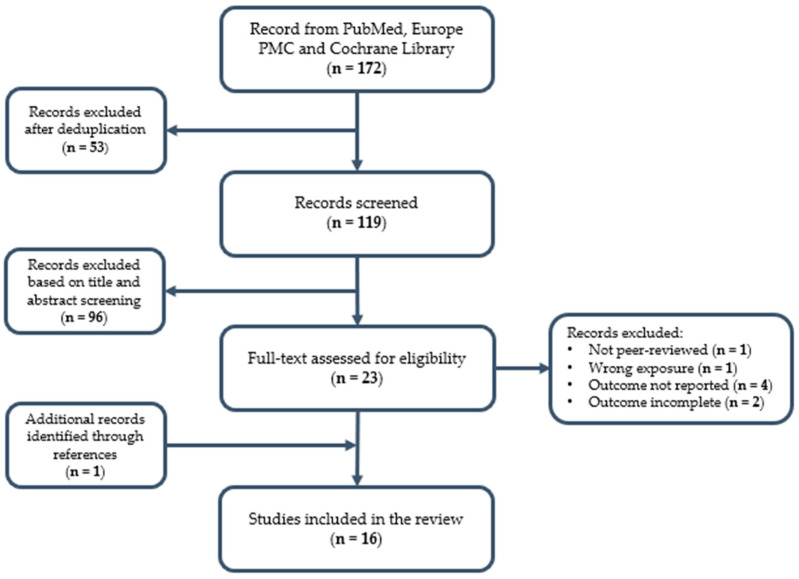
Flow diagram of the studies included in the review.

Due to heterogeneity in study design and outcome reporting, the results were summarised descriptively. The selection process is summarised in Figure 1.

## 3. Results

### 3.1. Single-Centre Retrospective Case Series

In total, 22 cases of maternal exposure to LCM throughout pregnancy (1 monotherapy, 21 polytherapy) were identified, resulting in 21 live births (95.5%) and 1 spontaneous abortion at 13 weeks of gestation (4.5%). Five patients experienced two pregnancies each. One pregnancy resulted from in vitro fertilisation. Median age at pregnancy was 32 years (range 25–42 years) and the majority of patients (50%) suffered from temporal lobe epilepsy (TLE) (11/22). Patient characteristics are summarised in Table 1.

Monotherapy with LCM at a 100 mg daily dose was administered in one patient, whereas all other patients received LCM as part of polytherapy (median dose of LCM 400 mg, range 100–600 mg). The most common combination was LCM with LEV (72.7%) (median dose of LEV 3000 mg, range 2000–3000 mg), followed by CBZ (22.7%) (median dose of CBZ 900 mg, range 300–1200 mg). One patient received a triple therapy consisting of LCM, LEV, and zonisamide (ZNS) (daily dose of ZNS 400 mg). LCM was started before conception in all but three women (86.4%) and was taken throughout the entire pregnancy without dose adjustment. Three WWE experienced focal-to-bilateral tonic–clonic seizures (fBTCS) during the first trimester of pregnancy, of whom two (9.1%) had discontinued pre-established LCM (100 mg) without medical consultation prior to pregnancy. LCM was re-established in both patients in the first trimester of pregnancy with seizure improvement in one and seizure freedom in the other patient. In one patient with drug-resistant TLE, LCM 200 mg was established as part of a triple therapy during the second trimester (19 weeks of gestation) due to frequent fBTCS with seizure improvement. Among the remaining patients, the majority (72.7%) remained seizure-free from all seizure types throughout the entire pregnancy. Consecutive serum levels with stable doses of LCM were taken in 18/22 pregnancies (81.8%) showing a median change in LCM levels of −17.7% (range −71.4%; +29.3%) with a decrease in 14/18 patients (77.8%; median decrease −21.35; range −3.1 to −71.4), and an increase in 3 patients (median increase 23.7; range 3.1 to 29.1). Due to the small sample size, no correlation between change in serum levels and seizure frequency could be made. Most women (86.4%) took folic acid supplementation during pregnancy at a median daily dose of 5 mg.

**Figure 2:**
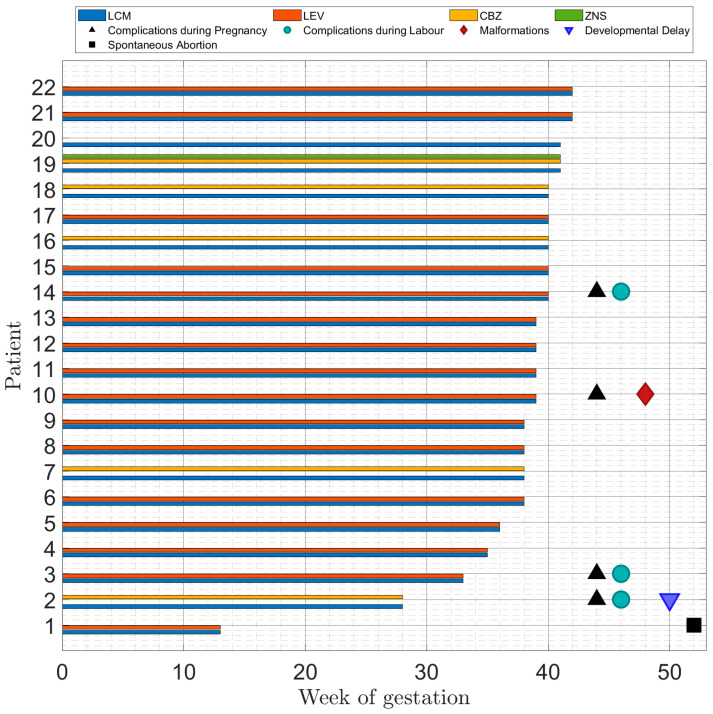
Lacosamide exposure and outcome of pregnancies.

Non-seizure-related complications during pregnancy or birth occurred in four pregnancies (18.2%) (Table 2, Figure 2). In a 38-year-old patient (#2) with frontal lobe epilepsy treated with LCM 500 mg and CBZ 1050 mg, severe preeclampsia led to an emergency caesarean section at 28 weeks of gestational age (birth weight 620 g, length 33 cm, APGAR-Score 6/8/8), with subsequent slight neurodevelopmental delay after twelve months. In a 34-year-old patient (#3) with TLE and treatment with LCM 200 mg and LEV 3000 mg, non-seizure-related placental abruption led to caesarean section at 32 weeks of gestation. The female infant (birth weight 2030 g, length 43 cm, APGAR-Score 6/9/9) was treated at the neonatal intensive care unit (NICU) with unremarkable neurocognitive development after twelve months. One spontaneous abortion at 13 weeks of gestation occurred in a 33-year-old patient receiving polytherapy with LCM 400 mg and LEV 3000 mg (#1). A previous pregnancy in this patient (#14) taking the same ASM regimen led to labour induction due to polyhydramnion and foetal arrhythmia at 40 weeks of gestation. A healthy female infant (birth weight 3180 g, length 53 cm, APGAR-Score 9/10/10) with unremarkable development after twelve months was subsequently delivered by uncomplicated vaginal birth.

One case of congenital malformation (#10) in the sense of an atrial septal defect was detected in a male newborn exposed to polytherapy with LCM 400 mg and LEV 3000 mg (4.8%). The mother suffered from gestational diabetes and hypertension during pregnancy and had experienced severe preeclampsia in a previous pregnancy while treated with LEV. Details on non-seizure-related pregnancy, labour and neonatal complications are illustrated in Figure 2 and Supplementary Table S1.

Altogether, one infant was born very preterm (at 28 weeks), and three other infants (#3, #4, and #5) were born moderate or late preterm (between 32 and 37 weeks), at a median gestational age of 34 weeks (range 28–36), with a median birth weight of 2682.5 g (range 620–3600 g) and a median length of 46.5 cm (range 33–53 cm). Four infants (18.2%) were small for gestational age (SGA) (<10th percentile) with a median birth weight of 2605 g (range 2405–2855), median length of 48 cm (range 46–50), and median head circumference of 33 cm (range 31.5–33).

Follow-up and developmental outcome data based on parental reports of age-appropriate developmental milestones were collected during in-person follow-up visits at 12 months. One patient relocated abroad and follow-up was therefore not available. No neurodevelopmental disorders were observed except in the very preterm infant born at 28 weeks (4.5%) as reported above. Twelve newborns were breastfed for at least six months (57.1%). The most common reported reason for not breastfeeding was insufficient milk production.

Although no correlation between LCM dose and congenital malformations, pregnancy complications, delivery complications, or neurodevelopmental outcomes was observed in this cohort, the study is limited by the small sample size. Therefore, given the low number of events, the results are presented descriptively.

Pregnancy, neonatal, and developmental outcomes are summarised in Table 2.

### 3.2. Comprehensive—Review of the Literature

In total, 16 studies reporting foetal outcomes following LCM exposure during pregnancy and/or breastfeeding were identified. An overview of the included studies is given in Table 3. Eight case reports, three case series, four cohort/observational studies and one pharmacovigilance report were included in this narrative review, altogether reporting data on 627 pregnancies with LCM (236 monotherapy, 391 polytherapy) with 632 available pregnancy outcomes (3 twin pregnancies and 1 triplet in the polytherapy group) [13,14,15,18,19,20,21,22,23,24,25,26,27,28,29,30]. These resulted in 514 live births (81.3%); 88.6% [209/236] with monotherapy and 77.0% [305/396] with polytherapy. One infant exposed to a combination therapy of LCM and CBZ died on day one due to an arteriovenous malformation of the liver and foetal hydrops [23]. MCMs were reported in 53/632 offspring (8.4%): 2.5% [6/236] of patients with monotherapy and 11.9% [47/396] of patients with polytherapy. Altogether, SGA was reported in 3.2% (20/632). An overview of the specific MCMs reported in the included studies is given in Supplementary Table S2.

#### 3.2.1. Case Reports and Case Series

The first case report on the use of LCM in pregnancy was published by Ylikotila et al. describing a 36-year-old woman suffering from status epilepticus (SE) due to cerebral venous thrombosis during the first trimester of pregnancy (8 weeks of gestation) [13]. Treatment included an ASM regimen with LEV, fosphenytoine and LCM, as well as general anaesthesia. After cessation of SE, a combination therapy with LEV 2000 mg and LCM 200 mg was maintained for the entire pregnancy. A healthy male infant (APGAR score 8/9/9), SGA (birth weight 2200 g), was delivered by planned caesarean section at 36 weeks without congenital malformations and unremarkable development at 7 months. Another case of new onset refractory status epilepticus (NORSE) in the second trimester of pregnancy (19 week of gestation), treated with LEV 2000 mg, OXC 900 mg and LCM 400 mg, LTG 100 mg, as well as general anaesthesia and immunotherapy, was reported by Karadjole et al. SE resolved after 26 days and the patient was continued on ASM polytherapy with LEV, LCM and OXC, as well as corticosteroids [29]. A healthy male infant was born by uncomplicated induced vaginal delivery at 38 weeks. No congenital malformations or immediate behavioural dysfunction were reported in the offspring; however, follow-up was limited to 6 weeks.

Early prospective evidence on the use of LCM in pregnancy is provided by the small case series of Lattanzi et al., who reported three infants exposed to either LCM monotherapy (two cases) or LCM in combination with LEV (one case) at a median dose of 400 mg (range 300–400 mg). Follow-up extended to 18–36 months showed favourable neurodevelopmental outcomes [18].

These findings were supported by Bosak et al., who prospectively reported four pregnancies with LCM monotherapy. Three resulted in healthy newborns who were breastfed and demonstrated normal developmental milestones at twelve months of age. One pregnancy ended in miscarriage at 7 weeks of gestational age [25]. Several additional retrospective case reports and small case series (nine polytherapy, four monotherapy), with no observation of teratogenicity or adverse early developmental outcomes, further strengthened the evidence for the safe use of LCM [14,15,19,21,22,24].

One retrospective case series (three LCM monotherapy, four polytherapy), focussed on changes in LCM levels throughout pregnancy. It described a significant decrease in dose-normalised concentrations (DNCs) of LCM that was most pronounced in the second and third trimesters. Among seven live births, two infants were delivered preterm (gestational age 24 weeks and 36 weeks), of which one died of sudden infant death syndrome (SIDS) at 5 months. No MCM were reported in all seven offspring; however, follow-up data on neurodevelopmental outcomes are lacking [20].

#### 3.2.2. Cohort and Observational Studies

Larger observational studies provide a more nuanced picture on the use of LCM during pregnancy and breastfeeding. Hoeltzenbein et al. reported outcomes from 65 pregnancies exposed to LCM (55 prospective, 10 retrospective) including 6 monotherapies (9.2%) and 59 patients on polytherapy (90.8%) [23]. While most pregnancies resulted in live births (66.2%) without major complications, three spontaneous abortions (4.6%), one stillbirth diagnosed with trisomy 18 (1.5%) and eight elective terminations of pregnancy (12.3%) were reported. MCMs were reported in four pregnancies (6.2%) with LCM polytherapy. Two infants showed cardiac abnormalities, of which a patent foramen ovale (PFO) and coarctation of the aorta after exposure to LEV and LTG were detected in one offspring and an atrial septum defect II after treatment with LCM and LEV in the other. One pregnancy exposed to VPA in the first trimester and LCM in the second trimester was terminated at 21 weeks of gestational age due to multiple malformations. A fourth offspring exposed to LCM and CBZ died one day after birth after partial resection of the liver because of a congenital arteriovenous liver malformation and foetal hydrops. Furthermore, there was one case of mild hydronephrosis and cryptorchidism in an offspring exposed to combination therapy of LCM, LEV, OXC and clobazam (CLB). Notably, this study also described the first reports of neonatal bradycardia potentially associated with in utero LCM exposure in three offspring, beginning on day 3 of life and resolving spontaneously by day 5. In addition, one unspecified cardiac arrhythmia was reported. Neonatal LCM levels were measured in two of the affected offspring and were within the therapeutic range.

In another retrospective observational study examining the use of newer-generation ASMs during pregnancy, Devin et al. reported on six LCM-exposed pregnancies (three monotherapy, three polytherapy) [28]. Pregnancy outcomes included five live births (83.3%) and one spontaneous abortion (16.7%). One neonate postnatally needed intensive care treatment (20%), and hyperbilirubinemia, sedation and feeding disturbances were reported in two offspring each (40%). One offspring was SGA (20%). No major or minor congenital malformations at birth were detected; however, no follow-up data on neurocognitive development are provided [28].

In a large population-based cohort study, Christensen et al. investigated the effects of prenatal exposure to ASMs on intrauterine foetal growth, including nine cases of LCM monotherapy [26]. No association between LCM exposure and SGA (mean birth weight 3567 g, adjusted mean difference 119 g) or microcephaly (mean head circumference 35.4 cm, adjusted mean difference 0.77) was detected. This study did not focus on congenital malformations or neurodevelopmental outcomes, with no follow-up data of exposed offspring available.

The largest cohort study on the risk of MCMs associated with monotherapy with newer ASMs was derived from the North American Antiepileptic Drug Pregnancy Registry [30]. No MCMs were detected in 88 offspring exposed to LCM monotherapy, whereas a risk of 2.2% (6/272) for MCM in LCM polytherapy was reported. Seven infants (8.0%) were born preterm, and eight neonates (9.1%) were SGA. No follow-up data on neurodevelopment of the offspring was provided.

#### 3.2.3. Pharmacovigilance Data

The most extensive data on LCM exposure during pregnancy are available from a pharmacovigilance report by Perucca et al., who analysed reports from the UCB Pharma global pharmacovigilance database, encompassing 202 prospectively collected pregnancies exposed to LCM (44 (21.5%) monotherapy; 158 (78.2%) polytherapy) [27]. Among the 204 reported outcomes (2 twin pregnancies with polytherapy), the overall proportion of live births was 84.1% (37/44) with LCM monotherapy and 76.3 (122/160) with polytherapy. Congenital malformations were reported in 2.3% (1/44) of pregnancies with LCM monotherapy and 6.9% (11/160) with polytherapy, all exposed to LCM throughout the first trimester of pregnancy. The single case of MCM with exposure to LCM monotherapy 100 mg was an offspring with ear malformations without associated hearing disorder. Among the eleven offspring with MCM under LCM polytherapy (three pregnancies ≥3 ASMs), two neural tube defects, two cardiac defects and two renal defects were described (for details, see Supplementary Table S1). Altogether, 15 infants (9.4%) were born preterm (10.8% [4/37] monotherapy, 9.0% [11/122] polytherapy). Four neonates (2.5%) (2.7% [1/37] monotherapy; 2.5% [3/122] polytherapy) were SGA.

Furthermore, 235 retrospectively collected cases of maternal exposure to LCM during pregnancy derived from pharmacovigilance data were also reported in the same study, including 76 monotherapies (32.3%) and 159 polytherapy cases (67.7%). The proportion of live births among 238 reported outcomes (1 twin pregnancy and 1 triplet in the polytherapy group) was comparable between monotherapy (77.1%) and polytherapy (74.1%). MCMs occurred in 6.6% (5/76) of patients with LCM monotherapy and 19.8% (32/162) with LCM polytherapy. Preterm delivery (5.1% monotherapy [3/59]; 15.0% polytherapy [18/120]) and SGA (1.7% monotherapy [1/59]; 4.2% polytherapy [5/120]) were more frequently reported with LCM polytherapy in this cohort.

In contrast to these mostly reassuring findings, recent analyses of the FDA Adverse Event Reporting System (FAERS) by Zeng et al. and Ji et al. have raised potential safety concerns regarding LCM exposure during pregnancy [32,33]. Using disproportionality analyses, these studies identified signals suggesting possible associations between LCM exposure and adverse foetal growth outcomes, congenital malformations, and developmental abnormalities. Because no exact data on the number of LCM exposed cases, details on course of pregnancy, number and type of MCM and neurodevelopment, as well as clinical case descriptions, were available in these studies, they were not included in this narrative review.

## 4. Discussion

The available evidence on LCM exposure during pregnancy and breastfeeding remains limited and heterogeneous. Most published data derive from small case series and observational cohorts, while prospective studies and data from pregnancy registries are scarce. Epilepsy pregnancy registries are the most important tool to systematically assess pregnancy outcomes and major congenital malformations (MCMs) in WWE exposed to ASMs during pregnancy and breastfeeding. However, newer ASMs are prescribed with caution until sufficient safety data concerning teratogenicity are available. Therefore, even years after marketing authorisation, only small numbers of LCM-exposed pregnancies (<100 WWE) from pregnancy registries have been published to date, and long-term data are still missing.

Moreover, firm safety conclusions for LCM itself are difficult to draw as most reported exposures occur in polytherapy. Interpretation across studies is further complicated by heterogeneous outcome definitions, absent comparator groups, variable follow-up durations, and reporting bias. Pharmacovigilance datasets are particularly prone to preferential reporting of severe or unusual outcomes. Consequently, reported rates of MCM are likely to be overestimated. Furthermore, pharmacovigilance reports often provide insufficient information to adjust for concomitant ASMs, dosing, and other clinically relevant factors.

To close this gap and aid clinicians when counselling WWE in pregnancy, we provide a comprehensive overview of all available data on LCM during pregnancy and/or breastfeeding. Moreover, our single-centre experience with LCM exposure during pregnancy and breastfeeding expands the current body of evidence.

In our single-centre retrospective case series, LCM exposure during pregnancy and breastfeeding, predominantly as part of polytherapy, was not associated with significant signals for adverse pregnancy outcomes, congenital malformations, delivery complications, or impaired early neurodevelopment. Although statistical power was limited by the small sample size, our findings are reassuring and consistent with previously published case series and cohort studies. The comparatively long follow-up of a minimum of one year did not reveal any neurocognitive impairment in the offspring.

Several limitations of this study must be acknowledged. First, the retrospective design made systematic assessment of relevant covariates, including genetic or familial predisposition to MCM, detailed obstetric risk factors, and other potential confounders, difficult. Second, only one patient in our cohort received LCM as monotherapy; therefore, our data largely reflect outcomes after exposure to LCM in polytherapy, which mirrors routine clinical practice in women with refractory epilepsy. In this context, teratogenic risk is critically dependent on the specific ASMs used in combination. LEV, the most common concomitant ASM in our cohort, has not been associated with an increased risk of MCM when used as monotherapy [34,35].

By including all available studies on LCM exposure during pregnancy in this narrative review, however heterogeneous they are, we aim to give a nuanced picture of LCM safety in pregnancy. However, a major source of bias is the retrospective design of many studies. Particularly, retrospective pharmacovigilance data, which account for one third of all reported cases, are prone to reporting bias and consequently may overestimate the rate of MCM. Analysing the rate of MCM in prospective cases and registry data only, an overall low rate of MCM of 4.2% (15/361), especially when used as monotherapy (0.7% [1/153]) compared with polytherapy (6.7% [14/208]), is reassuring regarding its safety during pregnancy. No specific MCM has been consistently attributed to LCM, with cardiac defects being most frequently reported (1.2%) after exposure to LCM polytherapy.

LCM was part of polytherapy in 391 pregnancies. As details on combination therapies were not provided in some studies and the sample size of different combinations was too small, we were not able to perform a subanalysis of different polytherapy combinations. Therefore, it remains difficult to assess the exclusive influence of LCM on MCMs and other outcome measures. However, our own data indicate that combination therapy of LCM and LEV is not associated with MCMs or impaired neurocognitive development. Furthermore, no correction for other potential confounders could be made due to insufficient clinical information provided in the original studies and the retrospective design of most studies.

In addition to the occurrence of MCM, LCM does not seem to be associated with an increased risk of spontaneous abortion, premature delivery, or clinically relevant impairment of intrauterine foetal growth. As follow-up duration varied substantially across studies and no standardised assessment of neurocognitive development in the offspring was performed, no clear statement on the impact of LCM on cognitive and behavioural function in the offspring can be made. However, no alarming intermediate behavioural disturbances (e.g., drinking weakness, somnolence) or neurodevelopmental dysfunction have been observed in children exposed to LCM during pregnancy and breastfeeding.

An important but unrecognised aspect of LCM is its cardiac safety profile. LCM prolongs the PR interval without affecting the QTc-interval or heart rate in adults, with a dose-dependent but generally clinically insignificant effect [36,37]. However, rare cases of atrioventricular (AV) block have been reported [31]. While these findings are overall reassuring in adults, their applicability to in utero exposure and neonatal populations remains uncertain. Importantly, neonatal cardiac effects following in utero LCM exposure have been reported by Hoeltzenbein et al. [23]. Furthermore, a second-degree AV block and cardiac arrest has been reported in a three-week-old neonate with seizures treated with LCM with resolution after discontinuation [38]. In our cohort, one patient required an emergency caesarean section due to abnormal foetal heart rate, which resolved spontaneously following delivery. These cases may suggest the potential vulnerability of the immature cardiac conduction system, warranting cardiac monitoring of exposed newborns especially in cases of prematurity, high maternal doses or polytherapy including other sodium channel blockers.

## 5. Conclusions

Overall, our single-centre retrospective case series reflects everyday clinical practice and, together with the available literature, does not indicate major safety concerns associated with LCM use during pregnancy or breastfeeding. LCM exposure was not associated with a high risk of adverse pregnancy outcomes, congenital malformations, or impaired early neurodevelopment. Our own study data and the provided summary of the literature add to the limited evidence of the use of LCM in pregnant WWE. Larger, well-designed prospective studies with standardised outcome measures, detailed documentation of concomitant antiseizure medications, and longer neurodevelopmental follow-up are needed to more clearly define the safety profile of lacosamide, particularly when used as monotherapy.

## Figures and Tables

**Table 1:** Demographical and clinical data of the patients (n = 22); n (%) or median (range).

Characteristics	
Maternal age at time of conception, years, median (range)	32 (25–40)
LCM dose at time of conception, milligrams, median (range)	400 (100–600)
Serum LCM concentration before pregnancy, µg/mL; median (range)	10.9 (0–17.7)
Serum LCM concentration during pregnancy, µg/mL; median (range)	8.7 (2.4–17.1)
LCM monotherapy	1/22 (4.5%)
Polytherapy	
Levetiracetam	16/22 (72.7%)
Carbamazepine	5/22 (22.7%)
Zonisamide	1/22 (4.5%)
Type of epilepsy	
Temporal lobe epilepsy	11/22 (50%)
Frontal lobe epilepsy	5/22 (22.7%)
Other focal epilepsy	5/22 (22.7%)
Generalised epilepsy	1/22 (4.5%)
fBTCS in the first trimester	3/22 (13.6%)
Folic acid intake ≥ 0.4 mg/day	19/22 (86.4%)
Smoking	2/22 (9.1%)
Use of other medications	4/22 (18.2%)

**Table 2:** Pregnancy, developmental and breastfeeding outcomes; n (%) or median (range).

Characteristics	
Live birth	21/22 (95.5%)
Spontaneous abortion	1/22 (4.5%)
Gestational week at birth	39 (28–42)
Very premature birth	1/21 (4.8%)
Moderate or late premature birth	3/21 (14.3%)
Pregnancy complications	
Preeclampsia	1/21 (4.8%)
Placental abruption	1/21 (4.8%)
Abnormal foetal heart rate	1/21 (4.8%)
Gestational diabetes	1/21 (4.8%)
Labor complications	3/21 (14.3%)
Sex of the child	
Male	13/21 (61.9%)
Female	8/21 (38.1%)
Head circumference, cm, median (range)	34.8 (29–37)
Weight, grams, median (range)	3060 (620–4160)
Size, cm, median (range)	50 (33–53)
Apgar Score	
Apgar 1	9 (6–10)
Apgar 5	10 (8–10)
Apgar 10	10 (8–10)
Breastfeeding for at least 6 months	12/21 (57.1%)
Neurodevelopmental delay at 12 Months	1/20 (5%)

**Table 3:** An overview of studies reporting on the safety of LCM during pregnancy and/or breastfeeding. Studies are presented in chronological order.

Study	Study Design	LCM-Exposure	LCM Sample Size	Follow-Up	Key Outcomes Related to LCM
Ylikotila et al. [13] (2015)	Case report	Polytherapy: LCM + LEV	1 pregnancy	At birth	No major/minor malformations, SGA
Lattanzi et al. [18] (2017)	Prospective case series	2 cases of monotherapy 1 case of polytherapy: LCM + LEV	3 pregnancies	Infancy and childhood	No major/minor malformations; breastfed; normal development at follow-up at 18–36 months
Kohn et al. [19] (2020)	Case report	Polytherapy: LCM + LEV	1 pregnancy	Infancy	No major/minor malformations; breastfed; developmental milestones at 6 months of age achieved
Landmark et al. [14] (2021)	Case report	Polytherapy: LCM + BRV + PER	1 pregnancy	Infancy	No major/minor malformations; developmental milestones at 12 months achieved
Zutshi et al. [20] (2021)	Retrospective case series	4 Polytherapy LCM + OXC or other ASMs, 3 Monotherapy	7 pregnancies	At birth	None of the infants showed major malformations
Fukushima et al. [21] (2021)	Case report	Polytherapy: LCM + CBZ + CLB	1 pregnancy	At birth	No complications during delivery; healthy offspring at birth
Dono et al. [31] (2022)	Case report	Polytherapy: LCM + LEV	1 pregnancy	At birth	No major/minor malformations
Hoeltzenbein et al. [23] (2023)	Observational study (prospective and retrospective)	6 cases of monotherapy 59 cases of polytherapy: LCM + other ASMs	65 pregnancies	At birth	3 spontaneous abortions, 1 stillbirth (trisomy 18); 1 infant died 1 day after birth, 8 pregnancies electively terminated 4 cases of MCMs + 1 mild hydronephrosis 3 cases of bradycardia + 1 unspecified cardiac arrhythmia
Cercos et al. [15] (2024)	Case report	Monotherapy	1 pregnancy	Infancy	No major/minor malformations; breastfed; no adverse outcomes at 12 months
Waack et al. [24] (2024)	Case report	Polytherapy: LCM + LEV + CLB	1 pregnancy	At birth	Delivered via planned caesarean section due to placenta previa; healthy offspring at birth
Bosak et al. [25] (2024)	Prospective case series	Monotherapy	4 pregnancies	Infancy	One miscarriage at 7 weeks of gestation; no congenital malformation; breastfed; developmental milestones at 12 months achieved
Christensen et al. [26] (2024)	Cohort study	9 cases of monotherapy	9 pregnancies	At birth	LCM monotherapy not associated with SGA
Perucca et al. [27] (2024)	Pharmacovigilance database analysis of prospective cases	44 cases of monotherapy 158 cases of polytherapy: LCM + other ASMs	202 pregnancies	30 days after birth	204 reported outcomes (2 twin pregnancies with polytherapy); live births 84.1% (37/44) monotherapy, 76.3% (122/160) polytherapy, MCM: 2.3% monotherapy (1/44); 6.9% polytherapy (11/160); SGA: 2.7% monotherapy (1/37), 2.5% polytherapy (3/122)
Perucca et al. [27] (2024)	Pharmacovigilance database analysis of retrospective cases *	76 cases of monotherapy 159 cases of polytherapy	235 pregnancies	Not stated	238 reported outcomes (1 twin and 1 triplet pregnancy with polytherapy) Live birth: 77.6% monotherapy (59/76); 74.1% polytherapy (120/162); MCM: 6.6% monotherapy (5/76), 19.8% polytherapy (32/162); SGA: 1.7% monotherapy (1/59); 4.2% polytherapy (5/120)
Devin et al. [28] (2025)	Observational retrospective study	3 cases of monotherapy 3 cases of polytherapy: LCM + other ASMs	6 pregnancies	At birth	No major/minor malformations, 1 spontaneous abortion, SGA (1 case); hyperbilirubinemia (2); sedation (2); feeding disturbance (2)
Karadjole et al. [29] (2025)	Case report	Polytherapy: LCM + BRV + OXC + CLB	1 pregnancy	At birth	No complications during delivery; healthy offspring at birth
Hernandez-Diaz et al. [30] (2025)	Cohort study	Monotherapy	88 pregnancies	At birth	No major/minor malformations at birth, seven infants (8.0%) were born preterm, and eight neonates (9.1%) were SGA.
Ji et al. [32] (2025) *	Pharmacovigilance data	13 cases of monotherapy 42 cases of polytherapy	55 pregnancies	Not mentioned	Analysis of FDA Adverse Events Reporting System (FAERS); ROR (Reporting Odds Ratio) for MCM monotherapy 0.9 (0.5–1.6), mono- + polytherapy 1.7 (1.2–2.2)

* Data not included in narrative review due to substantial reporting bias and insufficient clinical information. Abbreviations: ASMs—antiseizure medications; MCMs—major congenital malformations; LCM—lacosamide; LEV—levetiracetam; BRV—brivaracetam; CBZ—carbamazepine; CLB—clobazam, OXC—oxcarbazepine; PER—perampanel; SGA—small for gestational age.

## Data Availability

Data available from the corresponding author on demand.

## Supplementary Materials

The following supporting information can be downloaded at: https://www.mdpi.com/article/10.3390/pharmacy14020058/s1, Table S1: Characteristics of patients who experienced adverse pregnancy, labour or neonatal outcomes in the single-centre case series; Table S2. Overview of specific congenital malformations under LCM exposure.

## Abbreviations

The following abbreviations are used in this manuscript:

AD	autism spectrum disease
ASMs	antiseizure medications
BRV	brivaracetam
CLB	clobazam
CBZ	carbamazepine
DNCs	dose-normalised concentrations
FAERS	FDA Adverse Event Reporting System
fBTCs	focal-to-bilateral tonic clonic seizures
ID	intellectual disability
MCMs	major congenital malformations
NICU	neonatal intensive care unit
NORSE	new onset refractory status epilepticus
LEV	levetiracetam
LCM	lacosamide
LTG	lamotrigine
OXC	oxcarbazepine
PER	perampanel
PFO	patent foramen ovale
SGA	small for gestational age
SIDS	sudden infant death syndrome
TLE	temporal lope epilepsy
TPM	topiramate
VPA	valproic acid
WWE	women with epilepsy
ZNS	zonisamide
