# Supplementary material for: Lacosamide Safety During Pregnancy and Breastfeeding: A Single-Centre Experience and Comprehensive Narrative Review
Source: Pharmacy (Basel). 2026 Apr 1;14(2):58. doi: 10.3390/pharmacy14020058 (PMC13119888; doi:10.3390/pharmacy14020058)
Supplement: Supplementary file 1 [file pharmacy-14-00058-s001.zip › pharmacy-4183371-supplementary.pdf]

Supp. Table S1. Characteristics of patients who experienced adverse pregnancy, labour or neonatal outcomes in the single centre case series.

Patient	Age (year)	LCM dose (mg/day)	Other ASM, dose (mg/day)	Seizure Occurrence /Type	Folic acid	Nicotine	Other medications	Pregnancy complications	Labour complications	Malformations	Developmental delay
**1**	33	400	LEV, 3000	no	no	yes	no	spontaneous abortion at 13. weeks	n/a	n/a	n/a
**2**	38	500	CBZ, 1050	no	yes	no	no	preeclampsia	preterm delivery at 28 weeks of gestation.	no	no
**3**	34	200	LEV, 3000	yes, focal	yes	no	no	placental abruption	preterm delivery at 33. weeks of gestation	no	no
**10**	42	400	LEV, 3000	no	yes	no	yes, methyldopa and insulin	gestational diabetes	no	no	no
**14**	30	300	LEV, 3000	no	yes	no	no	fetal arrhythmia	emergency caesarean section at 40.weeks of gestation	no	no

Abbreviations: ASM- antiseizure medication; LCM - lacosamide; LEV- levetiracetam; CBZ carbamazepine

Supp. Table S2. Overview of specific congenital malformations under LCM exposure

Specific Malformation	Number of Cases (n)	LCM Monotherapy	LCM Polytherapy	Concomitant ASM(s) and its dose (mg)	Study
**Arteriovenous liver malformation**	1	-	✓	CBZ , n/a	Hoeltzenbein et al., 2023
**Atrial septal defect**	2	-	✓	LTG , 700	Perucca et al., 2024
		-	✓	LEV, n/a	Hoeltzenbein et al., 2023
**Cardiac septal defect**	1		✓	LTG, n/a	Perucca et al., 2024
**Cleft palate**	1		✓	BRV, 100; ESL, 800	Perucca et al., 2024
**Coarctation of the aorta**	2	-	✓	LTG, n/a	Hoeltzenbein et al., 2023
				LTG , 700	Perucca et al., 2024
**Congenital cystic kidney disease**	1	-	✓	TPM, 250	Perucca et al., 2024
**Cytogenetic abnormality**	1	-	✓	LEV, 2000	Perucca et al., 2024
**Ear malformation**	1	-	✓	n/a	Perucca et al., 2024
**Gastroschisis**	1		✓	PER, 2000	Perucca et al., 2024
**Hydronephrosis und cryptorchidism**	1	-	✓	CLB; OXC; LEV	Hoeltzenbein et al., 2023
**Multiple fetal malformation (spina bifida, cleft palate, radial aplasia)**	1	-	✓	CLB, 40-60; LTG, 100; LEV, 8000; PHB, 50-300; PHT, 600; ZNS 800	Perucca et al., 2024
**Polydactyly**	1	-	✓	LEV, 3000	Perucca et al., 2024
**Pulmonary hypoplasia**	1	-	✓	TPM, 250	Perucca et al., 2024
**Renal aplasia**	1	-	✓	VPA, n/a	Perucca et al., 2024
**Spina bifida**	1	-	✓	CBZ, 1200	Perucca et al., 2024
**Tuberous sclerosis complex**	1	-	✓	CLN, LEV, ZNS, n/a	Perucca et al., 2024

ASMs- antiseizure medications ; BRV – brivaracetam; CBZ – carbamazepine; CLB - clobazam; CLN – clonazepam; ESL – eslicarbazepine; LTG- lamotrigine; LEV-levetiracetam; PER – perampanel; PHB- phenobarbital; OXC – oxcarbazepine; PHT- phenytoin; TPM – topiramate; VPA- valproic acid; ZNS - zonisamid
